# First Trimester Mean Platelet Volume, Neutrophil to Lymphocyte Ratio, and Platelet to Lymphocyte Ratio Values Are Useful Markers for Predicting Preeclampsia

**DOI:** 10.31486/toj.21.0026

**Published:** 2021

**Authors:** Süleyman Cemil Oğlak, Şeyhmus Tunç, Fatma Ölmez

**Affiliations:** ^1^ Department of Obstetrics and Gynecology, Health Sciences University, Gazi Yaşargil Training and Research Hospital, Diyarbakır, Turkey; ^2^ Department of Obstetrics and Gynecology, Health Sciences University, Kanuni Sultan Süleyman Training and Research Hospital, Istanbul, Turkey

**Keywords:** *Hypertension–pregnancy-induced*, *inflammation*, *preeclampsia*, *pregnancy*, *systemic inflammatory response*

## Abstract

**Background:** Preeclampsia complicates 2% to 8% of all pregnancies. Systemic inflammatory response (SIR) markers are widely used in the diagnosis of many inflammatory diseases and in the prediction of complicated pregnancies. This study examined the diagnostic value of SIR markers during the first trimester of pregnancy to predict preeclampsia development.

**Methods:** This retrospective case-control study was conducted from January 2020 to May 2020. We included 94 patients diagnosed with mild preeclampsia, 107 patients diagnosed with severe preeclampsia, and 100 normotensive pregnant patients as controls. We obtained the first trimester (6 to 14 weeks) complete blood cell counts for all patients. We used a receiver operating characteristic curve to evaluate the cutoff, sensitivity, and specificity values.

**Results:** First trimester mean platelet volume (MPV), neutrophil to lymphocyte ratio (NLR), and platelet to lymphocyte ratio (PLR) values were significantly higher in patients who developed preeclampsia in later pregnancy weeks. The optimal cutoff value for MPV was 10.65 fL, with a sensitivity of 63.7% and a specificity of 65.0%. The best predictor for preeclampsia was NLR at an optimal cutoff value of 4.12, with a sensitivity of 82.1% and specificity of 62.0%. At a cutoff value of 131.8, PLR predicted preeclampsia with a sensitivity rate of 65.0% and a specificity rate of 60.2%.

**Conclusion:** The results of this study suggest that first trimester MPV, NLR, and PLR values are clinically useful markers in the prediction of preeclampsia. The increased first trimester values of MPV, NLR, and PLR also indicate that inflammation may play a crucial role in preeclampsia pathogenesis.

## INTRODUCTION

Preeclampsia is a severe syndrome marked by new-onset hypertension after 20 weeks of gestation accompanied by proteinuria or various maternal end-organ dysfunctions, leading to maternal and fetal-neonatal morbidity and mortality.^1^ This hypertensive disorder complicates 2% to 8% of all pregnancies and is responsible for more than 70,000 maternal deaths and 500,000 fetal deaths worldwide every year.^1,2^ Preeclampsia is a systemic disorder that may cause multiple maternal organ dysfunctions, including renal, hepatic, pulmonary, neurologic, and hematologic complications.^3,4^ Also, preeclampsia may result in fetal complications, including oligohydramnios, growth restriction, preterm birth, placental abruption, and perinatal death.^5,6^

Preeclampsia is considered a 2-stage disorder; defective trophoblast invasion and spiral artery remodeling failure are postulated as the primary step responsible for preeclampsia pathogenesis.^1^ Consequently, impaired uteroplacental blood flow results in hypoxia. The placenta's oxidative stress causes the release of factors, including proinflammatory cytokines, antiangiogenic agents, exosomes, and cell-free fetal DNA, into the maternal circulation that further trigger endothelial dysregulation and increased vascular permeability.^7,8^

Because of this pathophysiologic cascade, various biomarkers have attracted interest in the second decade of the 21st century for their potential utility in preeclampsia prediction as early as the first trimester.^9^ Identifying patients with increased preeclampsia risk and predicting this disease early improve outcomes and enable prophylaxis with aspirin and early intervention.^10^ Studies have demonstrated that hyperactivation of inflammatory cells and immunologic responses of lymphocytes and neutrophils occur by releasing proinflammatory biomarkers driving endothelial dysregulation.^11,12^

The phrase *low-grade inflammation* is used to explain situations identified by a slightly increased immune cell count and elevated proinflammatory protein values in cases with no sign of an inflammatory disease.^13^ Systemic inflammatory response (SIR) markers, including red cell distribution width (RDW), plateletcrit (PCT), mean platelet volume (MPV), platelet distribution width (PDW), platelet to lymphocyte ratio (PLR), and neutrophil to lymphocyte ratio (NLR), are readily available as systemic inflammation markers from a complete blood count (CBC) and are widely used in the diagnosis of many inflammatory diseases and in the prediction of complicated pregnancies.^11,14,15^ However, published results regarding the association between preeclampsia and SIR markers remain controversial with conflicting reports. Whereas some studies demonstrated these markers to be useful in predicting preeclampsia, other studies did not.^13,16^

This study investigated the diagnostic value of SIR markers during the first trimester of pregnancy to predict preeclampsia development.

## METHODS

This retrospective case-control study was conducted at the Department of Obstetrics and Gynecology at Diyarbakır Gazi Yaşargil Training and Research Hospital from January 2020 to May 2020. The ethics committee of the hospital approved the study (28.04.2020/462).

The study groups consisted of hospitalized and delivered mild or severe preeclamptic patients between 24 to 40 weeks of gestation. Control group patients were randomly selected from among healthy normotensive pregnant females without proteinuria, hospitalized for delivery at ≥37 weeks of pregnancy during the same period. We matched the 3 groups for age and body mass index (BMI). The exclusion criteria for study participants were as follows: patients with inadequate data, multiple gestations, molar pregnancy, prepregnancy BMI ≥30 kg/m^2^, a history of recurrent miscarriages or infertility, known thrombophilia or any other medical condition needing chronic drug treatment, previous pregnancy with gestational hypertensive disorders, complicated pregnancies (preterm delivery, preterm premature rupture of membranes, chorioamnionitis, intrauterine fetal demise, gestational diabetes mellitus, intrahepatic cholestasis of pregnancy), patients with malignancies, the use of acetylsalicylic acid and smoking during pregnancy, and fetuses with chromosomal or morphologic abnormalities.

During the study period, we identified 107 patients with mild preeclampsia, identified 116 patients with severe preeclampsia, and randomly selected 113 healthy pregnant controls. After applying the exclusion criteria and withholding patients with missing medical reports, 94 patients remained in the mild preeclampsia group, 107 patients remained in the severe preeclampsia group, and 100 patients remained in the control group.

We used the American College of Obstetricians and Gynecologists criteria for preeclampsia definition.^17^ We diagnosed mild preeclampsia after 20 weeks of gestation when systolic blood pressure (BP) was ≥140/90 mmHg or diastolic BP was ≥90 mmHg in at least 2 measurements made 4 hours apart in a patient with a previously normal BP and proteinuria (≥300 mg/24-hour urine collection, protein to creatinine ratio of ≥0.3 mg/g, or a dipstick reading of 2+ protein). In the absence of proteinuria, patients with gestational hypertension were diagnosed with preeclampsia if they presented with any of the following severe features: thrombocytopenia (platelet count of <100,000/mL), renal insufficiency (serum creatinine level of >1.1 mg/dL or a doubling of the serum creatinine concentration in the absence of renal disease), liver dysfunction (increased blood levels of transaminases to twice normal concentration), new-onset headache (unresponsive to medication and not accounted for by other diagnoses) or visual disturbances, and pulmonary edema.

We defined severe preeclampsia as systolic BP of ≥160 mmHg or diastolic BP of ≥110 mmHg on 2 occasions at least 4 hours apart in a patient with a previously normal BP and the presence of proteinuria (≥300 mg/24-hour urine collection, protein to creatinine ratio of ≥0.3 mg/g, or a dipstick reading of 2+ protein). In the absence of proteinuria, patients with gestational hypertension were diagnosed with severe preeclampsia if they presented with any of the following severe features: thrombocytopenia, liver dysfunction, renal insufficiency, pulmonary edema, new-onset headache, or visual disturbances.

Age, gestational week, gravida, parity, and BMI were obtained by examining patients’ medical records. BP data were obtained just prior to labor induction or cesarean section. Gestational week was examined by sonographic measurement and confirmed according to last menstrual period and first trimester ultrasound. We obtained the first trimester (6 to 14 weeks) CBC counts for all patients. When more than one CBC result was accessible, we recorded the closest result to 6 weeks of gestation for statistical analysis.

CBC values were measured with a Mindray BC 6800 (Mindray Bio-Medical Electronics Co), an automatic blood counting device that uses laser and impedance measurement techniques. Hemoglobin, white blood cell count (WBC), neutrophil count, lymphocyte count, platelet count, RDW, MPV, PDW, and PCT values were extracted from patients' medical records. We calculated the NLR by dividing the neutrophil count by the lymphocyte count. We calculated the PLR by dividing the platelet count by the lymphocyte count.

IBM SPSS software, version 21.0 (IBM Corp) for Microsoft Windows was used for statistical evaluation of our research data. Measured variables are presented as mean ± SD, and categorical variables are presented as numbers and percentages. We used the Kolmogorov-Smirnov test to determine if the numerical data matched the normality distribution. We used the *t* test to compare the normally distributed data. Mann-Whitney *U* test with Bonferroni correction and Kruskal-Wallis H test were used to compare the non-normally distributed data. We used the chi-square test to compare the qualitative variables. Differences were considered statistically significant at *P*<0.05. We used a receiver operating characteristic (ROC) curve to evaluate the cutoff, sensitivity, and specificity values. The cutoff value was estimated by using the Youden Index.

## RESULTS

We included 94 patients diagnosed with mild preeclampsia, 107 patients diagnosed with severe preeclampsia, and 100 normotensive pregnant patients as controls. Patients’ demographic characteristics and clinical features are presented in Table 1. We found no significant differences among the 3 groups in terms of maternal age, gravidity, parity, and gestational week at the screening. The mean systolic and diastolic BPs were significantly higher in the mild and severe preeclampsia groups vs healthy controls.

**Table 1.** Demographic Characteristics and Clinical Features by Group

Variable	Control Group, n=100	Mild Preeclampsia Group, n=94	Severe Preeclampsia Group, n=107	*P_1_* Value (Control vs Mild)	*P_2_* Value (Mild vs Severe)	*P_3_* Value (Control vs Severe)
Age, years	27.4 ± 6.1	28.3 ± 7.4	28.7 ± 6.8	0.288	0.314	0.209
Gravidity	2.79 ± 1.69	3.13 ± 2.15	3.24 ± 2.07	0.427	0.592	0.303
Parity	1.59 ± 1.57	1.70 ± 1.75	1.88 ± 1.99	0.894	0.879	0.736
Weeks of gestation at the screening	7.4 ± 1.2	7.2 ± 1.1	7.4 ± 1.3	0.553	0.492	0.861
Systolic blood pressure, mmHg	102.1 ± 9.2	131.9 ± 15.8	164.2 ± 15.9	**<0.001**	**<0.001**	**<0.001**
Diastolic blood pressure, mmHg	62.7 ± 6.9	92.9 ± 8.1	112.1 ± 10.0	**<0.001**	**<0.001**	**<0.001**

Notes: Values (other than *P* values) are mean ± SD. Blood pressure data were obtained just prior to labor induction or cesarean section.

First trimester SIR marker values of the participants are shown in Table 2. First trimester hemoglobin, RDW, lymphocyte count, PDW, and PCT values were comparable among all 3 groups. First trimester WBC counts were significantly higher in the mild and severe preeclampsia groups than in healthy controls (*P*<0.001 and *P*<0.001, respectively). First trimester neutrophil count was significantly lower in the control group than in the mild and severe preeclampsia groups (*P*=0.003 and *P*<0.001, respectively). However, first trimester WBC and neutrophil counts were similar between the mild and severe preeclampsia groups. First trimester platelet counts were significantly higher in the mild and severe preeclampsia groups vs the control group (*P*=0.015 and *P*=0.004, respectively). First trimester MPV value was significantly lower in healthy controls than in the mild and severe preeclampsia groups (*P*<0.001 and *P*<0.001, respectively). However, no significant difference was found regarding first trimester platelet count and MPV value between the mild and severe preeclampsia group (*P*=0.352 and *P*=0.505, respectively).

**Table 2.** First Trimester Systemic Inflammatory Response Marker Values by Group

Variable	Control Group, n=100	Mild Preeclampsia Group, n=94	Severe Preeclampsia Group, n=107	*P_1_* Value (Control vs Mild)	*P_2_* Value (Mild vs Severe)	*P_3_* Value (Control vs Severe)
Hemoglobin, g/dL	11.7 ± 1.4	12.2 ± 1.5	11.9 ± 1.7	0.226	0.280	0.392
Red cell distribution width, %	15.2 ± 2.3	14.5 ± 1.7	14.9 ± 2.3	0.074	0.066	0.117
White blood cell count, mm^3^ × 10^3^	9.7 ± 1.7	11.2 ± 2.9	11.6 ± 2.9	**<0.001**	0.243	**<0.001**
Neutrophil count, × 10^3^/μL	6.8 ± 1.7	8.2 ± 3.1	8.6 ± 3.5	**0.003**	0.235	**<0.001**
Lymphocyte count, × 10^3^/μL	2.0 ± 0.5	2.0 ± 0.6	2.1 ± 0.8	0.863	0.691	0.735
Platelet count, mm^3^ × 10^3^	242.5 ± 57.5	259.7 ± 48.5	266.7 ± 70.6	**0.015**	0.352	**0.004**
Platelet distribution width, %	16.2 ± 1.4	16.5 ± 0.3	16.4 ± 0.3	0.079	0.126	0.099
Mean platelet volume, fL	10.3 ± 1.3	10.6 ± 2.3	11.2 ± 1.4	**<0.001**	0.505	**<0.001**
Plateletcrit, %	0.2 ± 0.06	0.2 ± 0.06	0.2 ± 0.07	0.429	0.463	0.386
Neutrophil to lymphocyte ratio	3.1 ± 1.1	4.3 ± 1.4	4.6 ± 1.8	**<0.001**	0.441	**<0.001**
Platelet to lymphocyte ratio	121.1 ± 27.4	137.1 ± 44.9	138.1 ± 38.2	**0.016**	0.988	**0.020**

Note: Values (other than *P* values) are mean ± SD.

First trimester NLR values were significantly higher in patients with mild and severe preeclampsia vs the control group (*P*<0.001 and *P*<0.001, respectively). Also, first trimester PLR values were significantly higher in patients with mild and severe preeclampsia than in healthy controls (*P*=0.016 and *P*=0.020, respectively). Nonetheless, first trimester NLR and PLR values were similar between the mild and severe preeclampsia groups (*P*=0.441 and *P*=0.988, respectively).

**Figure 1.**
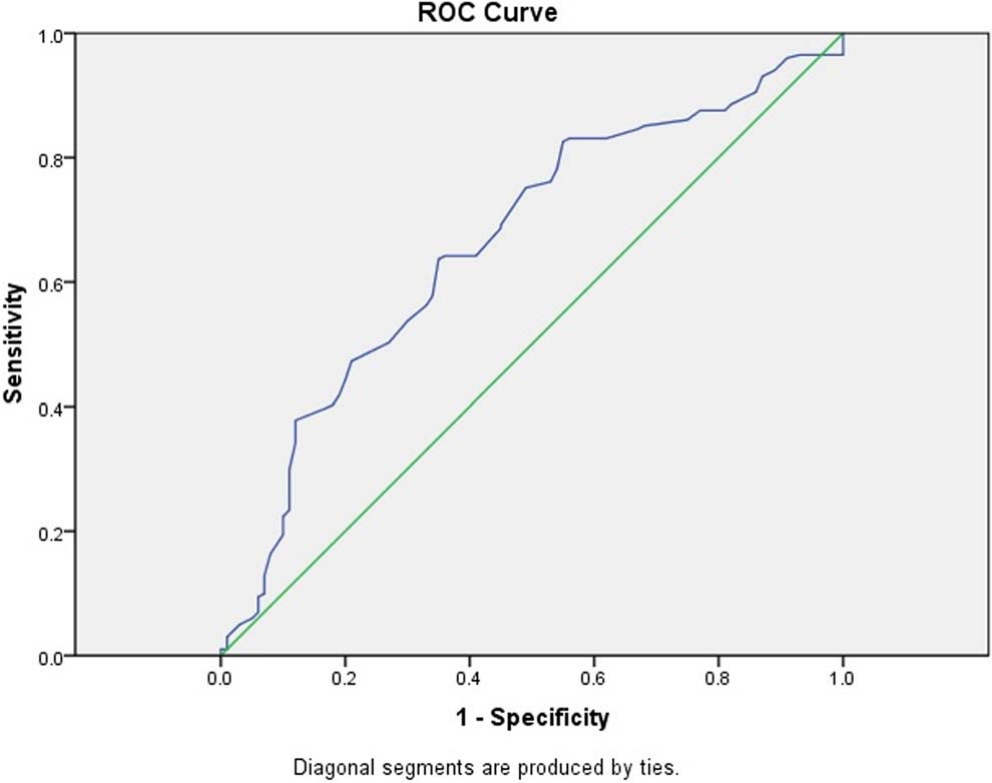
Receiver operating characteristic (ROC) curve for first trimester mean platelet volume value for predicting preeclampsia. The area under the curve is 0.663 (95% CI 0.598-0.728).

**Figure 2.**
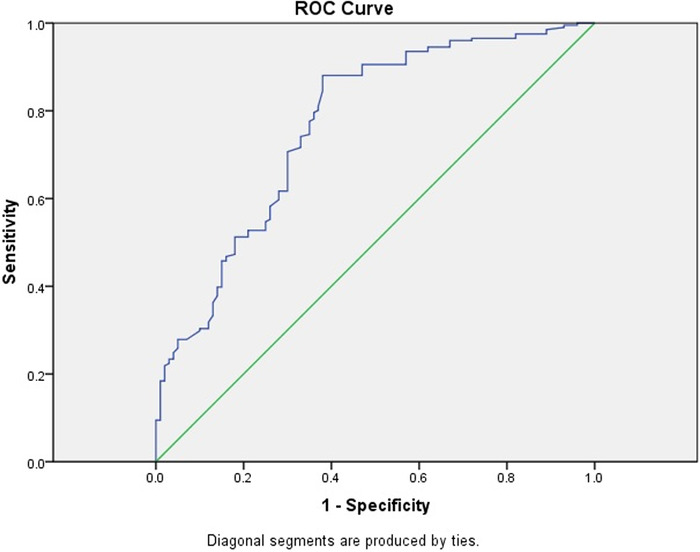
Receiver operating characteristic (ROC) curve for first trimester neutrophil to lymphocyte ratio value for predicting preeclampsia. The area under the curve is 0.767 (95% CI 0.709-0.826).

**Figure 3.**
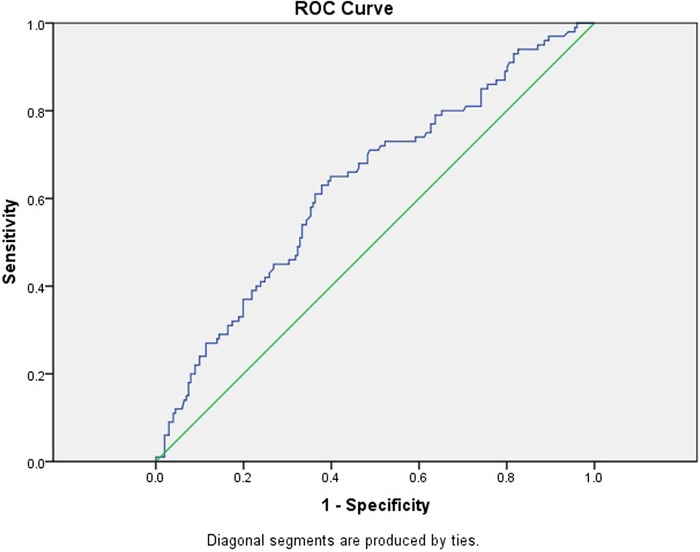
Receiver operating characteristic (ROC) curve for first trimester platelet to lymphocyte ratio value for predicting preeclampsia. The area under the curve is 0.631 (95% CI 0.565-0.698).

We used ROC curves to derive cutoff values of MPV, NLR, and PLR to predict preeclampsia (Table 3). The optimal cutoff value for MPV was 10.65 fL, with a sensitivity of 63.7% and a specificity of 65.0% (Figure 1). The best predictor for preeclampsia was NLR at an optimal cutoff value of 4.12, with a sensitivity of 82.1% and specificity of 62.0% (Figure 2). At a cutoff value of 131.8, PLR predicted preeclampsia with a sensitivity rate of 65.0% and a specificity rate of 60.2% (Figure 3).

**Table 3.** Area Under the Receiver Operating Characteristic (ROC) Curve of Mean Platelet Volume (MPV), Neutrophil to Lymphocyte Ratio (NLR), and Platelet to Lymphocyte Ratio (PLR)

			95% CI		
Variable	ROC	Standard Error	Lower	Upper	*P* Value
MPV	0.663	0.033	0.598	0.728	**<0.001**
NLR	0.767	0.030	0.709	0.826	**<0.001**
PLR	0.631	0.034	0.565	0.698	**<0.001**

## DISCUSSION

The current study demonstrated that first trimester MPV, NLR, and PLR values were significantly higher in patients who developed preeclampsia in later pregnancy weeks. However, these markers were not predictive in discriminating the severe preeclampsia cases from the mild cases.

Chronic inflammation has a central role in the pathogenesis of preeclampsia. During a healthy pregnancy, the balance between T helper 1 (Th1) and Th2 immune cells and their immune responses is crucial for maintaining gestation.^18,19^ A healthy pregnancy is thought to be a state with Th2 predominance, which provides an immunotolerant setting for fetal rejection prevention.^20^ However, in preeclamptic patients, placental ischemia as a result of shallow trophoblast invasion increases proinflammatory CD4+ T cells and reduces regulatory T helper cells.^19^ Th1/Th2 balance is disrupted, and a shift toward the Th1 response causes a chronic proinflammatory state with Th1 predominance.^21^ The ischemic placenta produces cytokines, including interleukin-6, tumor necrosis factor alpha, and soluble fms-like tyrosine kinase 1 (sFlt-1), and stimulates peripheral T cell activation that contributes to hypertension development by inducing B cells responsible for agonistic angiotensin II type 1 and endothelin-1 receptor autoantibodies.^22^ Understanding the mechanism underlying this pathophysiologic stage supports research about screening tests determining oxidative stress, endothelial dysfunction, and inflammatory markers for preeclampsia prediction.^23^ C-reactive protein, fibrinogen, alpha-1 antitrypsin, angiotensinogen, interleukins, alpha-1-acid glycoprotein, and ceruloplasmin are the acute phase inflammatory proteins that are elevated in patients with preeclampsia.^24^ Also, Akolekar et al reported that screening by the combination of maternal characteristics, mean arterial pressure, uterine artery pulsatility index, serum pregnancy-associated plasma protein A, and placental growth factor detected 54% of all cases of preeclampsia, <40% of cases of term preeclampsia, and 96% of cases of preeclampsia requiring delivery before 34 weeks of gestation.^25^ In their study, Rolnik et al^26^ included pregnant patients at high risk for preterm preeclampsia following the Akolekar et al criteria. Rolnik and colleagues indicated that administration of low-dose acetylsalicylic acid to these patients reduced the risk of preterm preeclampsia but did not reduce the incidence of term preeclampsia.^26^ Therefore, an effective method for predicting preeclampsia in the first trimester does not yet exist.

The diagnostic value of SIR markers such as NLR, PLR, PDW, MPV, PCT, and RDW in many diseases such as coronary artery disease, autoimmune diseases, inflammatory diseases, and malignancies has already been shown.^27,28^ However, the few studies focusing on preeclampsia have conflicting results on the association between the value of these markers in the first trimester and increased risk of preeclampsia development.^13,16^ We planned our study to evaluate whether these markers, which can be quickly obtained from the CBC, changed in patients before preeclampsia.

High RDW values are thought to reflect increased inflammation and oxidative stress, but the mechanism has not been fully established.^29^ Sen-Yu et al examined RDW values in the second trimester and reported that RDW values were higher in pregnant patients who developed preeclampsia in the third trimester.^30^ However, Çintesun et al stated that RDW levels did not differ between preeclamptic patients and healthy pregnant patients.^31^ We found no association between first trimester RDW values and subsequent preeclampsia development in our study.

In addition to their primary function in hemostasis, platelets are efficient immune modulators and effectors.^32^ Endothelial dysfunction induces vasoconstriction and platelet adhesion and aggregation, triggering coagulation and resulting in hypoxic damage to the endothelium.^33^ Thrombocyte consumption in the maternal peripheral circulation stimulates bone marrow production. The younger platelets produced at this stage are larger than the older ones and show a strong tendency toward aggregation.^34^ Therefore, the number, volume, and function of platelets change, and platelet turnover demonstrates an increase in preeclamptic maternal vasculature.^34,35^ Platelet count, PDW, MPV, and PCT are regarded as platelet activation markers.^36^ Tzur and Sheiner and Gezer et al reported high first trimester platelet counts in patients who develop preeclampsia.^37,38^ Kirbas et al found that while platelet count, MPV, and PCT values were similar among patients in the first trimester, PDW values were higher in the patients who later developed preeclampsia.^13^ Mannaerts et al stated that among platelet markers, only MPV was significantly increased before the 20th week of pregnancy in patients who subsequently developed preeclampsia vs healthy individuals.^39^ In our study, first trimester platelet count and MPV values were significantly higher in patients who developed preeclampsia than in the control group. This result suggests that platelet activation may have a crucial role in the pathogenesis of inflammation in preeclampsia. However, PDW and PCT values were comparable between the groups.

The interaction between platelets and vascular endothelium induces the release of inflammatory substances (chemokines, adhesion proteins, growth factors), and these substances cause leukocyte migration and adhesion.^40^ Canzoneri et al reported that the total leukocyte count was significantly increased in preeclamptic patients vs healthy pregnant females.^41^ The increased total leukocyte count was primarily attributable to the rise in neutrophil count. Our study found that WBC count and neutrophil count were significantly higher in patients who developed preeclampsia in the later weeks of gestation vs the controls. Kirbas et al demonstrated that first trimester NLR values were significantly higher in patients who subsequently developed preeclampsia vs healthy pregnant patients, and PLR values were significantly higher in patients who developed severe preeclampsia vs the mild preeclampsia group and the control group.^13^ Moreover, Kirbas et al reported that pregnant patients with high first trimester NLR values were significantly more likely to develop severe preeclampsia. Mannaerts et al reported that first trimester NLR and PLR values were similar in patients who would develop preeclampsia and healthy individuals.^39^ Gezer et al indicated that high PLR and NLR values during the first trimester are objective predictors of preeclampsia and could be used for early preeclampsia diagnosis.^38^ Likewise, our study demonstrated that first trimester NLR and PLR values were significantly higher in patients with established preeclampsia. However, these values were not useful in discriminating patients with severe preeclampsia from those with mild cases. Therefore, we suggest that enhanced inflammation and platelet activation already exist in the early gestation weeks, and first trimester NLR and PLR values might serve as a predictor of preeclampsia.

This study's major strength is that we examined first trimester CBC results to predict subsequent preeclampsia, parameters evaluated by a minority of previous research. Also, we stratified the patients with preeclampsia into mild and severe groups.

A limitation of this study is its retrospective design, with the limitations of such studies. Also, the change in NLR and PLR values during the course of the disease is unknown. Another limitation is the absence of levels of proinflammatory cytokines, including sFlt-1 and soluble endoglin, that have been previously identified with preeclampsia. A study that correlates the results of SIR markers with these cytokines may provide more insight into preeclampsia prediction.

## CONCLUSION

The results of this study suggest that first trimester MPV, NLR, and PLR values are clinically useful markers in the prediction of preeclampsia. The increased first trimester values of MPV, NLR, and PLR also indicate that inflammation may play a crucial role in preeclampsia pathogenesis.

## Notes

*This article meets the Accreditation Council for Graduate Medical Education and the American Board of Medical Specialties Maintenance of Certification competencies for Patient Care and Medical Knowledge.*
